# Supplementary material for: The Correlation between the Vascular Calcification Score of the Coronary Artery and the Abdominal Aorta in Patients with Psoriasis
Source: Diagnostics (Basel). 2023 Jan 11;13(2):274. doi: 10.3390/diagnostics13020274 (PMC9858225; doi:10.3390/diagnostics13020274)
Supplement: Supplementary file 1 [file diagnostics-13-00274-s001.zip › diagnostics-2103148-supplementary.pdf]

diagnostics

MDPI

Supplementary Materials

Table S1: CACS and AACS differences across subcategories.

Variables	Category	CACS				AACS			
		N	Mean	Median	p value	N	Mean	Median	p value
Noncalcified plaque	Negative	73	138.71	0.00	> 0.05	55	1128.58	206.33	> 0.05
	Positive	10	83.12	0.00		9	1689.36	1163.18	
Treatment	None	5	85.86	0.00	> 0.05	3	134.09	0.00	> 0.05
	Topical therapy	48	200.38	0.00		42	1300.49	295.35	
	PhotoTopical therapy	6	67.74	10.73		3	1038.43	1296.78	
	Oral therapy	3	8.21	0.00		3	1764.93	0.00	
	Anti-TNFalpha	8	12.10	0.00		6	325.37	18.15	
	Anti-IL17A	2	0.00	0.00		0			
	Anti-IL12/23	6	0.00	0.00		3	72.51	0.00	
Anti-TNFalpha treatment	Without	70	149.69	0.00	> 0.05	54	1178.71	211.93	> 0.05
	With	8	12.10	0.00		6	325.37	18.15	
Anti-IL17A treatment	Without	72	139.15	0.00	> 0.05	60	1093.38	202.55	N/A
	With	6	0.00	0.00		0			
Biologic Treatment	Anti-TNFalpha	8	12.10	0.00	> 0.05	6	325.37	18.15	> 0.05
	Anti-IL17A	6	0.00	0.00		3	72.51	0.00	
	Anti-IL12/23	2	0.00	0.00		0			
CHD	Negative	77	133.44	0.00	> 0.05	60	1238.31	211.93	> 0.05
	Positive	6	113.62	30.32		4	744.47	815.57	
Hyperlipidemia	Negative	39	178.35	0.00	> 0.05	28	1198.82	159.82	> 0.05
	Positive	44	90.93	0.00		36	1214.15	343.23	
Smoking	Negative	52	153.75	0.00	> 0.05	39	1574.58	455.34	> 0.05
	Positive	31	95.54	0.00		25	634.72	114.53	
Alcohol	Negative	47	103.87	0.00	> 0.05	36	977.25	163.61	> 0.05
	Positive	36	168.75	0.00		28	1503.41	545.68	
Metabolic Syndrome	Negative	28	110.40	0.00	> 0.05	25	925.03	37.33	> 0.05
	Undefined	30	213.00	0.00		20	1421.84	250.86	
	Positive	21	44.86	0.00		19	1353.35	455.34	
PSOfamilyHistory	Negative	81	134.18	0.00	> 0.05	62	1227.63	250.86	> 0.05
	Positive	2	44.20	44.20		2	581.59	581.59	

Table S2: CACS, AACS and categorical variable correlation.

Variables	Spearman correlation					
	CACS			AACS		
	N	Correlation coefficient r_{ho}	p value	N	Correlation coefficient r_{ho}	p value
Age	83	0.537	< 0.0001	64	0.681	< 0.0001
Age Onset	80	0.312	0.005	62	0.448	< 0.0001
Duration	79		> 0.05	61		> 0.05
PASI before treatment	83		> 0.05	64		> 0.05
PASI before CT	70		> 0.05	56		> 0.05
Comobidity (-/+)	83	0.385	< 0.0001	64	0.276	0.027

CACS				64	0.603	< 0.0001
Plaque Number	83	0.765	< 0.0001	64	0.520	< 0.0001
Noncalcified plaque (-/+)	83		> 0.05	64		> 0.05
Calcified plaque (-/+)	83	0.919	< 0.0001	64	0.517	< 0.0001
Stenosis Number	83	0.688	< 0.0001	64	0.455	< 0.0001
Stenosis Severity	31	0.509	0.003	25		> 0.05
Suffered Vessel Distribution	34	0.532	0.001	28	0.374	0.05
Total Cholesterol	82	-0.310	0.005	64	-0.277	0.027
Triglycerides	82	-0.227	0.041	64		> 0.05
HDL-Cholesterol	73		> 0.05	61		> 0.05
LDL-Cholesterol	77	-0.288	0.011	63		> 0.05
BMI	83		> 0.05	64		> 0.05
CRP	83		> 0.05	64		> 0.05
Fasting glucose	77	0.309	0.006	63	0.251	0.048
HbA1c	77		> 0.05	64	0.271	0.030
Systolic blood pressure	83	0.231	0.036	64	0.286	0.022
Diastolic blood pressure	83		> 0.05	64		> 0.05
Hypertension (-/+)	83	0.217	0.048	64	0.366	0.003
Diabetes (-/+)	83		> 0.05	64	0.269	0.031
Hyperlipidemia (+/-)	83		> 0.05	64		> 0.05
CHD (-/+)	83		> 0.05	64		> 0.05
CVD (-/+)	83	0.294	0.007	64		> 0.05
Smoking (-/+)	83		> 0.05	64		> 0.05
Alcohol (-/+)	83		> 0.05	64		> 0.05
Suita score	83		> 0.05	64		> 0.05
Framingham risk score	79		> 0.05	63	0.274	0.030
CHD after 10 years	74	0.279	0.016	61	0.388	0.002
Coronary family history (-/+)	83	-0.217	0.049	64		> 0.05
Psoriasis family history (-/+)	83		> 0.05	64		> 0.05
Biologics treatment (-/+)	78	-0.282	0.012	60		> 0.05
Anti IL-12/23 treatment (-/+)	78	-0.225	0.048	60		> 0.05

Table S3 (a–b): Data description during follow-up period.

	N	Minimum	Maximum	Mean	Standard deviation
1st CCTA – 2nd CCTA interval (month)	15	0	45	19	11
1st AbCT – 2nd AbCT interval (month)	20	0	83	32	26

(a)

Variables	N	Median	25 – 75 percentile		Range	
			Lower	Upper	Minimum	Maximum
PASI before treatment	64	10.00	5.50	17.75	1.20	53.40
PASI score 1	57	8.40	3.20	16.20	0.00	53.40
PASI score 2	14	0.20	0.00	2.00	0.00	10.40
CACS 1	64	0.00	0.00	76.04	0.00	1804.00
CACS 2	15	1.93	0.00	370.71	0.00	1575.20

AACS 1	64	250.86	0.00	1423.21	0.00	7580.57
AACS 2	20	124.25	10.93	1233.11	0.00	10790.07
1st visit – 1st CCTA interval (month)	64	1	0	6	0	83
1st CCTA – 1st AbCT interval (month)	64	0	0	2	0	24
CACS1-2 progression (calcium score/month)	15	0.00	0.00	2.56	-1.26	21.12
AACS1-2 progression (calcium score/month)	20	2.11	0.00	20.19	-73.82	95.05

(b)

Table S4 (a–c): PASI, CACS and AACS progression test.

Variables	Pair number	p value	
PASI before Treatment – PASI 1	57	< 0.0001	Pair t-test
PASI 1 – 2	14	> 0.05	
CACS 1 – 2	15	> 0.05	
AACS 1 – 2	20	< 0.0001	
CACS progression – AACS progression	3	> 0.05	
CACS progression – AACS progression		> 0.05	Man-Whitney test

(a)

Variables	Spearman rank correlation					
	Absolute CACS progression			Absolute AACS progression		
	N	Correlation coefficient r_{ho}	p value	N	Correlation coefficient r_{ho}	p value
Age	15		> 0.05	20	0.513	0.021
Age of onset	15		> 0.05	18	0.474	0.047
CACS 1	15		> 0.05	20	0.550	0.012
AACS 1	15		> 0.05	20	0.559	0.010
Triglycerides	15		> 0.05	20	-0.501	0.024
LDL-Cholesterol	15	-0.516	0.049	19		> 0.05

(b)

Variables		Relative CACS progression			Relative AACS progression		
		N	Median	p value	N	Median	p value
Comorbidity	Negative	12	0.00	> 0.05	14	0.0049	0.041
	Positive	3	0.009		6	0.0246	
CVD	Negative	13	0.00	> 0.05	16	0.0084	0.029
	Positive	2	0.0045		4	0.0258	

(c)

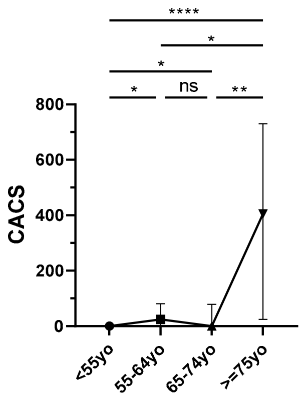

(a)

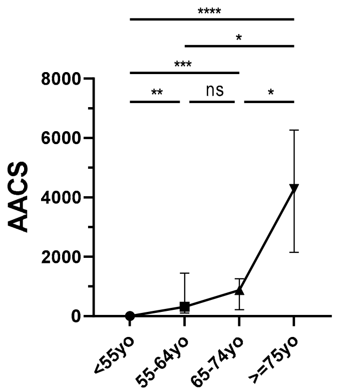

(b)

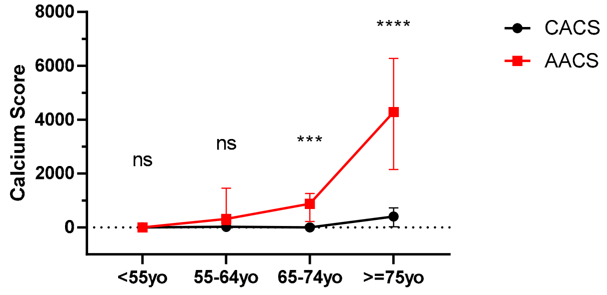

(c)

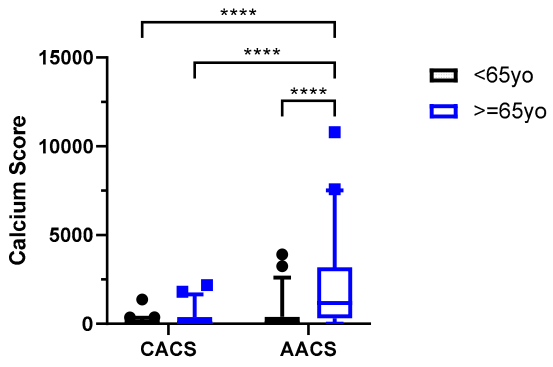

(d)

Figure S1 (a–d): CACS and AACS among different age group.

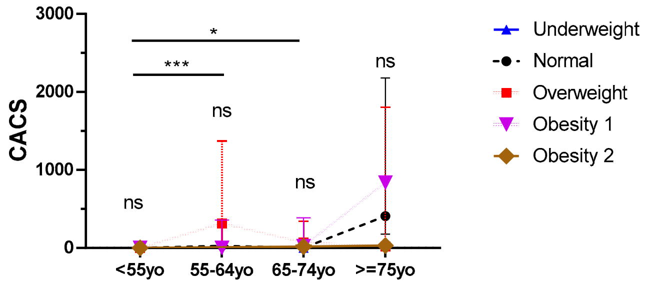

(a)

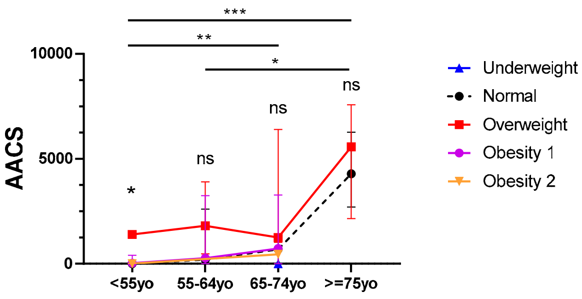

(b)

Figure S2 (a–b): CACS and AACS in different BMI categories and age group.

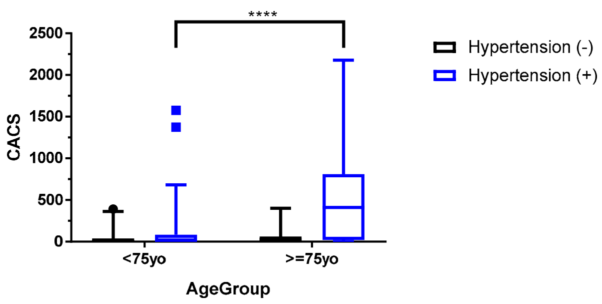

(a)

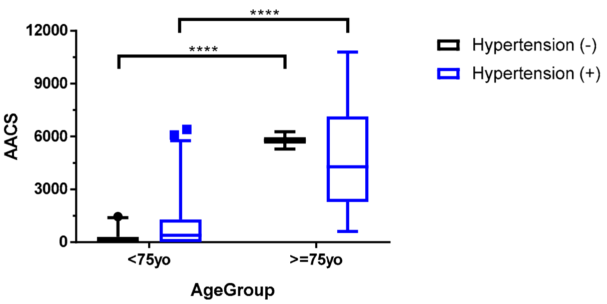

(b)

Figure S3 (a–b): CACS and AACS in patients with psoriasis with or without hypertension.

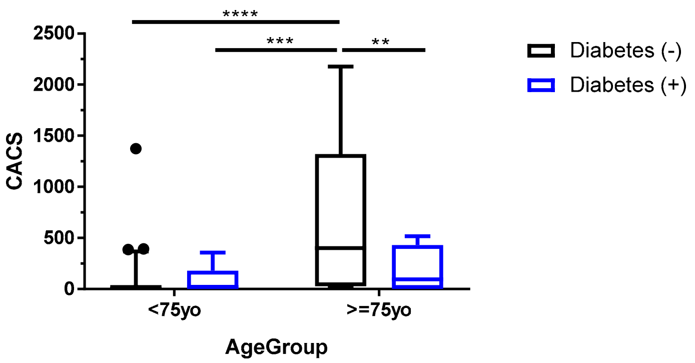

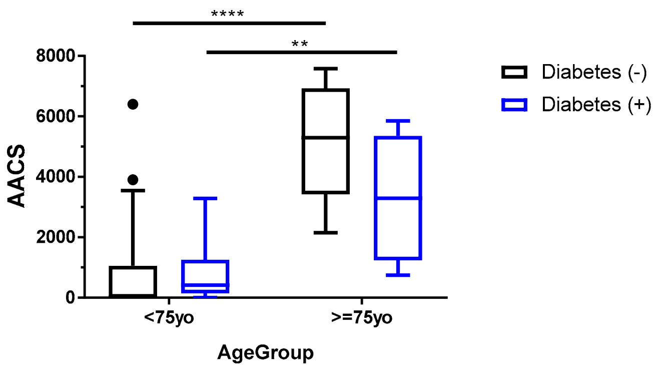

(a) (b)

Figure S4 (a–b): CACS and AACS in patients with psoriaisis with or without diabetes.

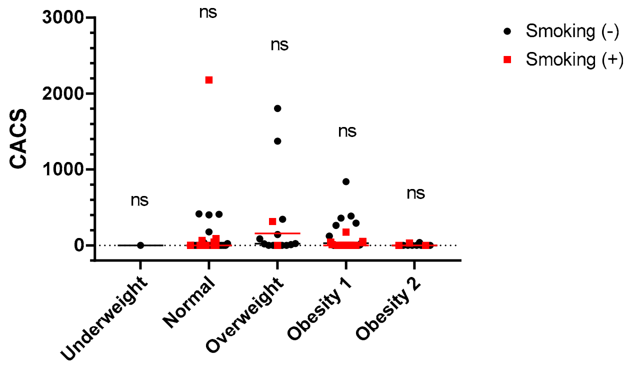

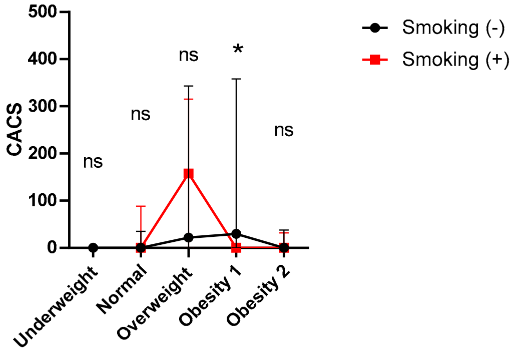

(a) (b)

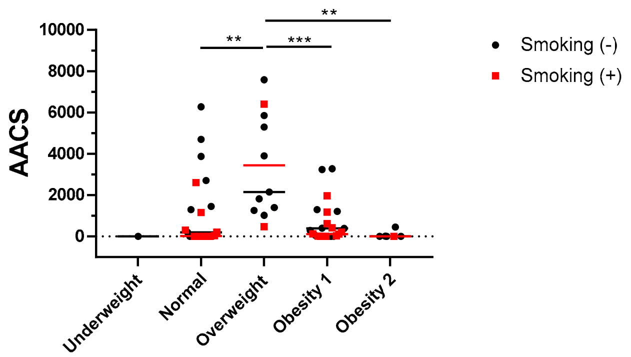

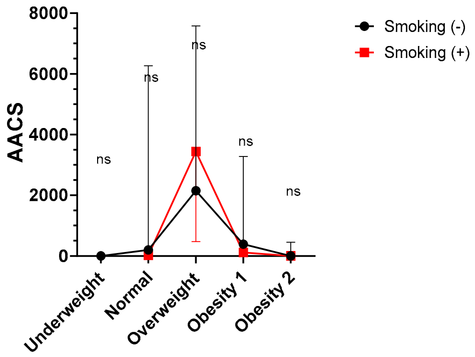

(c) (d)

Figure S5 (a–d): CACS and AACS in patients with psoriasis who do or do not smokein the context of BMI classification.
